# GC–MS analysis of the ruminal metabolome response to thiamine supplementation during high grain feeding in dairy cows

**DOI:** 10.1007/s11306-018-1362-8

**Published:** 2018-05-08

**Authors:** Fuguang Xue, Xiaohua Pan, Linshu Jiang, Yuming Guo, Benhai Xiong

**Affiliations:** 1 State Key Laboratory of Animal Nutrition, Institute of Animal Science, Chinese Academy of Agricultural Sciences, Beijing, 100193 China; 2 State Key Laboratory of Animal Nutrition, Institute of Animal Science, Chinese Agricultural University, Beijing, China; 3 Beijing Key Laboratory for Dairy Cow Nutrition, Beijing University of Agriculture, Beijing, China

**Keywords:** Thiamine, SARA, Metabolomics, Pyruvate metabolism, Dairy cows

## Abstract

**Introduction:**

Thiamine is known to attenuate high-concentrate diet induced subacute ruminal acidosis (SARA) in dairy cows, however, the underlying mechanisms remain unclear.

**Objectives:**

The major objective of this study was to investigate the metabolic mechanisms of thiamine supplementation on high-concentrate diet induced SARA.

**Methods:**

Six multiparous, rumen-fistulated Holstein cows were used in a replicated 3 × 3 Latin square design. The treatments included a control diet (CON; 20% starch, dry matter basis), a SARA-inducing diet (SAID; 33.2% starch, dry matter basis) and SARA-inducing diet supplemented with 180 mg of thiamine/kg of dry matter intake (SAID + T). On d21 of each period, ruminal fluid samples were collected at 3 h post feeding, and GC/MS was used to analyze rumen fluid samples.

**Results:**

PCA and OPLS-DA analysis demonstrated that the ruminal metabolite profile were different in three treatments. Compared with CON treatment, SAID feeding significantly decreased rumen pH, acetate, succinic acid, increased propionate, pyruvate, lactate, glycine and biogenic amines including spermidine and putrescine. Thiamine supplementation significantly decreased rumen content of propionate, pyruvate, lactate, glycine and spermidine; increase rumen pH, acetate and some medium-chain fatty acids. The enrichment analysis of different metabolites indicated that thiamine supplementation mainly affected carbohydrates, amino acids, pyruvate and thiamine metabolism compared with SAID treatment.

**Conclusions:**

These findings revealed that thiamine supplementation could attenuate high-concentrate diet induced SARA by increasing pyruvate formate-lyase activity to promote pyruvate to generate acetyl-CoA and inhibit lactate generation. Besides, thiamine reduced biogenic amines to alleviate ruminal epithelial inflammatory response.

**Electronic supplementary material:**

The online version of this article (10.1007/s11306-018-1362-8) contains supplementary material, which is available to authorized users.

## Introduction

Subacute ruminal acidosis (SARA) is an important nutritional metabolic disease in high yielding dairy cows because of the increased consumption of concentrates and highly-fermentable forages (Valente et al. 2017). The effects of SARA include decreased dry matter intake and lower milk yield (Enemark 2008), ruminal pH decrease, accumulation of biogenic amines and volatile fatty acids (VFAs) (Sato 2015), rumen epithelial damage (McCann et al. 2016) and laminitis (Plaizier et al. 2008). Since SARA leads to considerable damage in dairy cows, it is necessary to find a suitable mitigation method to attenuate SARA. Interestingly, our previous study revealed that thiamine supplementation could attenuate high-concentrate diet induced SARA by decreasing ruminal lactate production and increasing ruminal pH value in rumen fluid (Pan et al. 2016). The possible reason was that thiamine supplementation promoted carbohydrate metabolism, since thiamine is the coenzyme of pyruvate dehydrogenase (PDH) and α-ketoneglutaric acid dehydrogenase (α-KGDHC) in carbohydrate metabolism (Miller et al. 1986; Karapinar et al. 2008). However, it was not clear how thiamine supplementation affected the ruminal nutrient metabolism systematically in dairy cows. Therefore, more research on metabolic profile changes is needed to reveal the role of thiamine in ruminal metabolism regulation.

Metabolomics is an innovative and high-throughput bioanalytical method and has been utilized for detecting rumen metabolite biomarkers in recent years. Ametaj et al. (2010) firstly used the metabolomics method to detect the changed compounds with increasing amount of dietary grain. They found that harmful compounds including methylamine, nitrosodime, thylamine and ethanol were increased in SARA cows. Additional metabolites that have been identified as markers of SARA are carbohydrates, biogenic amines and amino acids (Saleem et al. 2012; Hua et al. 2017; Zhang et al. 2017). Therefore, the metabolomics method was chosen in this study to reveal whether thiamine supplementation affects carbohydrates, biogenic amines and amino acids metabolism or other metabolism pathways in order to interpret the metabolic mechanism of thiamine supplementation on high-concentrate diet induced SARA.

## Materials and methods

### Animals, experimental design and dietary treatments

Animal care and experimental procedures were operated in accordance with the Chinese guidelines for animal welfare and approved by Animal Care and Use Committee of the Chinese Academy of Agricultural Sciences.

Six Chinese Holstein dairy cows in second parity fitted with 10-cm ruminal cannulas (Bar Diamond, Parma, ID) were allocated to a replicated 3 × 3 Latin square design. Three periods were included and each experimental period consisted of 21 days (a 18-days adaptation period, followed by a 3-days period used for data and sample collection). Treatments included a control diet (CON; 20% starch, DM basis), SARA-induced diet (SAID, 33.2% starch, DM basis), and SARA induced diet supplemented with 180 mg of thiamine/kg of DMI (SAID + T). Details of ingredient analysis and chemical composition of dietary ingredients are given in Supplementary Table 1.

### Rumen fluid sampling

On the last day of each period, rumen contents were sampled from cranial, caudal, dorsal, and ventral sites of rumen at 3 h after the morning feeding. Collected samples were strained through four layers of cheesecloth to obtain rumen fluid. Rumen fluid was divided into two parts. One part was processed to analyze the pH value, the concentration of lactate, volatile fatty acids (VFA) and thiamine content. The other part was put into liquid nitrogen immediately after adding a stabilizer and then stored at − 80 °C for further analysis of the metabolome by GC–MS. The lactate concentration in rumen fluid was measured using enzymatic methods by commercial kits (A019-2, Nanjing Jiancheng Bioengineering Institute, Nanjing, China; technical parameters: recovery rate, 99%; CV, 1.7%; sensitivity < 0.1 mmol/L; detection range, 0–6 mmol/L) at 530 nm according to the manufacturer’s instructions. Individual and total VFA (TVFA) in aliquots of ruminal fluid were determined by gas chromatograph (GC-2010, Shimadzu, Kyoto, Japan).

### Metabolomics analysis

Agilent 7890 gas chromatograph system coupled with a Pegasus HT time-of-flight mass spectrometer (LECO, St,Joseph, MI) were used to conduct GC/MS analyses of samples. One hundred microliter of each sample was firstly mixed with 370 µL of solvents composed of 350µL methanol and 20 µL l-2-chlorophenylalanine (0.1 mg/mL stocked in dH_2_O), then the mixture were vortexed for 10 s and centrifuged for 15 min at 12,000 rpm, 4 °C. The supernatant (0.34 mL) was transferred into a fresh GC–MS glass vial, and 12 µL supernatant of each sample was taken and pooled as a quality control (QC) sample.

All samples were firstly dried in a vacuum concentrator without heating and then incubated for 20 min at 80 °C after adding 55 µL of methoxy amination reagent (20 mg/mL dissolved in pyridine) into each sample. Seventy-five microliter of BSTFA reagent (1% TMCS, v/v) was added to each sample then all samples were incubated for 1 h at 70–80 °C. Subsequently 10 µL Fatty Acid Methyl Ester (FAMEs) (Standard mixture of fatty acid methyl esters, C8–C16:1 mg/mL; C18–C24:0.5 mg/mL in chloroform) was adding into each sample after all samples were cooled to the room temperature. After added all reagents, each sample was mixed well for GC–MS analysis.

One microlitre of the analyte was injected into a DB-5MS capillary column coated with 5% diphenyl cross-linked 95% dimethylpolysiloxane (30m × 250 µm inner diameter, 0.25 µm film thickness; J&W Scientific, Folsom, CA, USA). One microliter of the analyte was injected in splitless mode. Helium was used as the carrier gas. The front inlet purge flow was 3 mL/min, and the gas flow rate through the column was 20 mL/min. The initial temperature was kept at 50 °C for 1 min, then raised to 320 °C at a rate of 10 °C/min. The temperature was kept for 5 min at 320 °C. The injection, transfer line, and ion source temperatures were 280, 280, and 220 °C respectively. The energy was − 70 eV in electron impact mode. The mass spectrometry data were acquired in full-scan mode with the m/z range of 85–600 at a rate of 20 spectra per second after a solvent delay of 366 s.

### Statistical analysis

Ruminal pH, VFA and thiamine content were analyzed using PROC MIXED of SAS 9.2 as shown in the following model: Y_ijklm_ = µ + T_i_ + P_j_ + S_k_ + C_l(Sk)_ + O_m_ + T_i_ × P_j_ + T_i_ × S_k_ + e^ijklm^, where Y_ijklm_ is the dependent variable, µ is the overall mean, Ti the fixed effect of treatment (i = 1–3), P_j_ is the fixed effect of period (j = 1–3), Sk is the random effect of Latin square (k = 1–2), C_l(Sk)_ is the random effect of cow nested in square (l = 1–6), Om is the fixed carryover effect from the previous period (O = 0 if period = 1), T_i_ × P_j_ is the interaction of treatment and period, T_i_ × S_k_ is the interaction between treatment and Latin square replicate, and e^ijklm^ is the random residual error. *p* value < 0.05 was considered to be significant and a tendency was considered at 0.05 ≤ *p* < 0.10.

For metabolomics data analysis, Chroma TOF 4.3X software of LECO Corporation and LECO-Fiehn Rtx5 database were used for raw peaks exacting, data baselines filtering and calibration of the baseline, peak alignment, deconvolution analysis, peak identification and integration of the peak area. The retention time index (RI) method was used in the peak identification, and the RI tolerance was 5000. In order to dislodge the noise data and conduct a better analysis for downstream data, all raw data was filtered by retaining the treatments with null value ≤ 50%.

Multivariate analysis including principal component analysis (PCA) and orthogonal correction partial least squares discriminant analysis (OPLS-DA) were conducted using SIMCA-P software (V 14.0, Umetrics, Umea, Sweden). Differentially expressed metabolites between two treatments were identified based on variable importance in projection (VIP) from OPLS-DA analysis and statistical analysis (VIP > 1 and *p* < 0.05). Kyoto Encyclopedia of Genes and Genomes (KEGG, http://www.genome.jp/kegg/) was conducted to view the enriched pathways of different metabolites. The Hierarchical clustering analysis (HCA) and heat map analysis for different metabolites were conducted using R package version 3.3.1.

## Results

### Ruminal pH, VFAs, lactate and thiamine

Data for rumen pH, ruminal VFAs, lactate and rumen thiamine content have been reported previously (Pan et al. 2017a). Briefly, mean ruminal pH in SAID treatment was 5.93 while in CON treatment was 6.49 and in SAID + T treatment was 6.15 (*p* < 0.001). The ruminal acetate and thiamine were significantly decreased in SAID feeding treatment compared with CON and SAID + T treatments (*p* < 0.05). Ruminal lactate and propionate were significantly increased in SAID treatment compared with the other two treatments (*p* < 0.05).

### Different ruminal metabolites between the CON vs SAID treatments and SAID vs SAIDT treatments

**Fig. 1:**
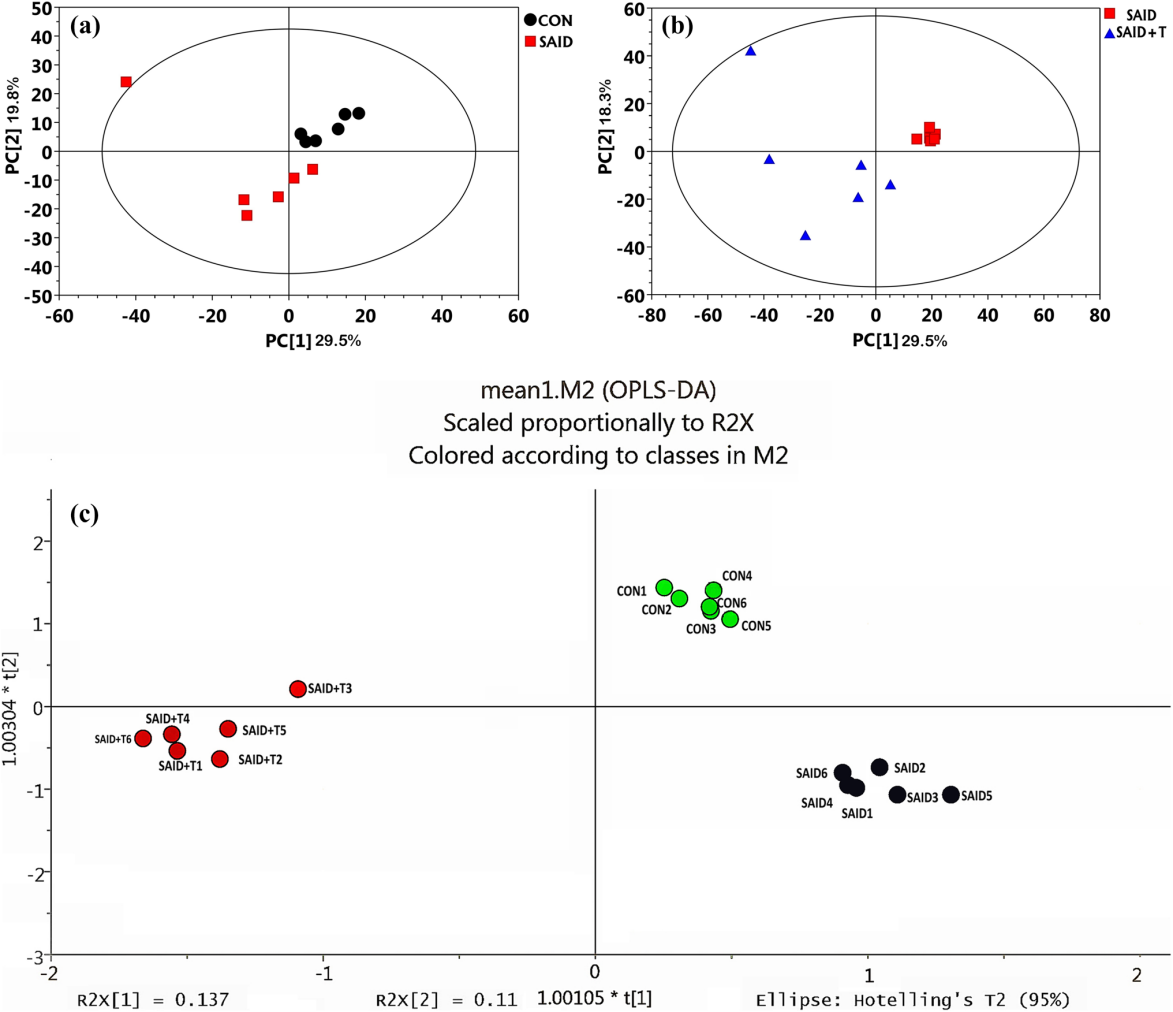
**a** Principal components analysis (PCA) of ruminal metabolites from cows (n = 6) fed a control diet (CON) and cows fed SARA induced diet (SAID); **b** PCA of ruminal metabolites from cows (n = 6) fed SAID and cows (n = 6) fed SARA induced diet with thiamine supplementation (SAID + T). **c** Orthogonal correction partial least squares discriminant analysis (OPLS-DA) of the ruminal metabolites from cows (n = 6) of the CON, the SAID and SAID + T treatments

Firstly, 534 peaks were detected with GC–TOF–MS and then, all raw data was filtered by retaining the treatments with null value ≤ 50%. Finally, 510 practicable peaks were obtained. After strict quality control and identification, 286 metabolites were obtained across all samples. They were mainly organic acids, fatty acids, carbohydrate, amino acids, purines and biogenic amines. For further analysis, PCA and OPLS-DA were conducted to analyze the ruminal metabolites among three treatments. As shown in Fig. 1a, the principal component analysis revealed that PCA axes 1 and 2 accounted for 29.5 and 19.8% and PCA axes 1 and 2 of Fig. 1b accounted for 29.5 and 18.3% of the total variation, respectively. As shown in Fig. 1c, OPLS-DA axes 1 and 2 accounted for 41.1 and 29.0% of the total variation, respectively. Samples of the three treatments could be separated clearly according to both PCA and OPLS-DA analysis.

**Fig. 2:**
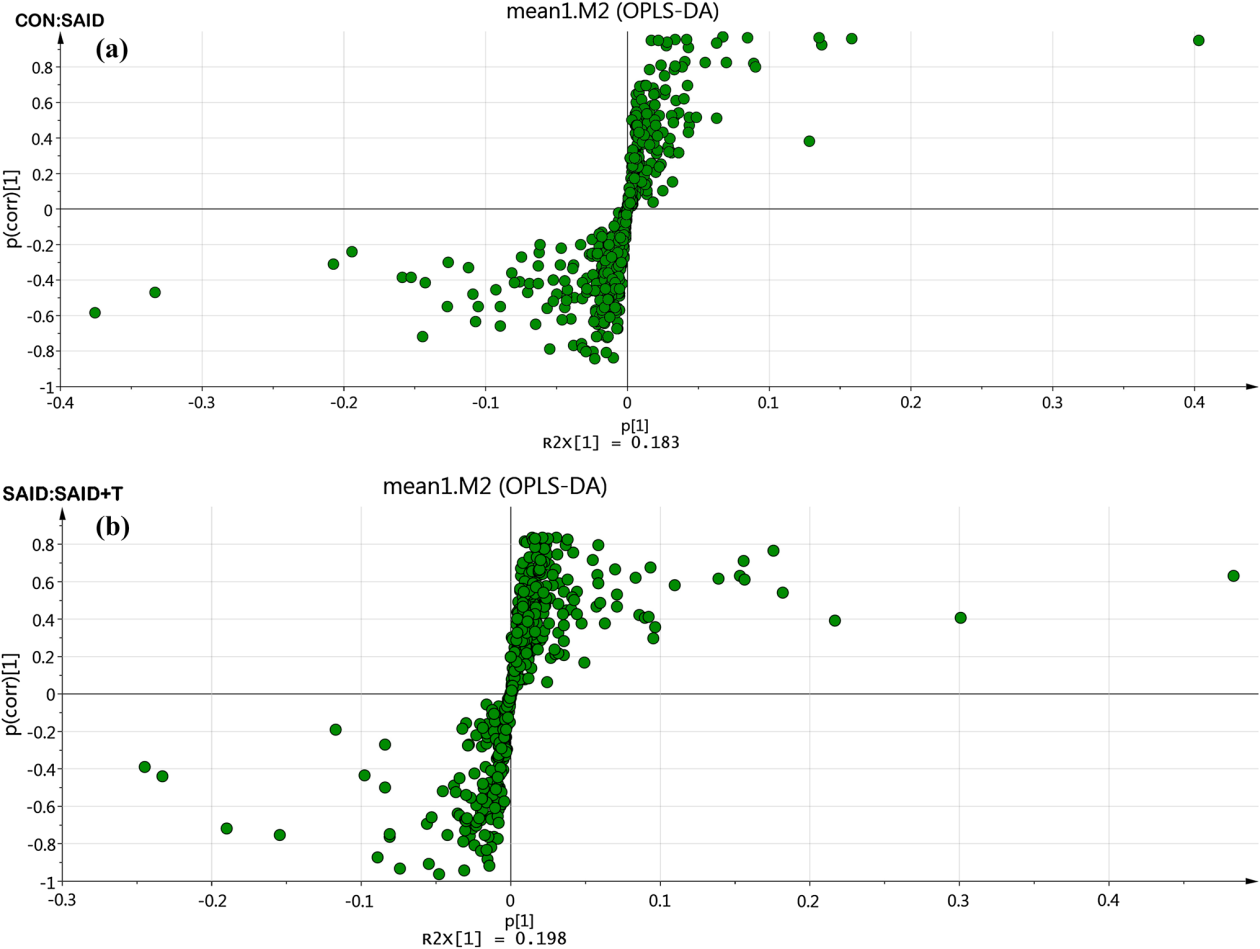
S-PLOT analysis of metabolites from cows (n = 6) fed control diet (CON) versus cows fed SARA induced diet (SAID) and metabolites from cows (n = 6) fed SAID versus cows (n = 6) fed SARA induced diet with thiamine supplementation (SAID + T)

As shown in Fig. 2a, b, based on OPLS-DA, an “S” plot was constructed. Pionts that located far from the axes represent metabolites that differ significantly between the two treatments. Results revealed that there were significantly changed metabolites between CON versus SAID and SAID versus SAID + T.

Combined with statistical analysis and the VIP value obtained from the OPLS-DA analysis, 59 differential metabolites between CON and SAID treatments, 23 between SAID and SAID + T treatments were identified (*p* < 0.05 and VIP > 1). These different metabolites are mainly carbohydrates, amino acids, organic acids and biogenic amines. Some typical and representative metabolites are represented in Tables 1 and 2.

**Table 1:** Different metabolites content in the rumen of dairy cows fed a control diet (CON) versus cows fed SARA induced diet (SAID)

Metabolites	RT	Mass	Similarity	VIP	Fold change	*p* value
Amino acids						
Glycine	11.22	174	944	1.784	0.497	0.016
Oxo proline	14.06	156	940	1.954	1.937	0.005
Glutamic acid	15.22	246	858	1.700	1.710	0.023
Nucleotides						
Thymidine	10.80	170	639	2.086	0.526	0.001
Hypoxanthine	17.37	265	814	1.075	3.690	0.000
Xanthine	19.48	353	803	1.273	3.650	0.002
Organic acids						
Caprylic acid	10.66	143	700	1.153	0.455	0.020
Phenylacetic acid	11.16	164	898	2.022	0.597	0.010
Pelargonic acid	11.99	215	933	1.538	0.821	0.022
Capric acid	13.29	117	631	1.405	0.849	0.039
Glutaric acid	12.56	147	875	1.809	1.616	0.012
Azelaic acid	17.23	55	937	1.774	1.627	0.004
2-Hydroxy butanoic acid	8.60	131	836	1.041	0.280	0.001
3-Hydroxy propionic acid	8.88	177	678	1.284	0.453	0.025
3-Hydroxybutyric acid	9.11	117	919	1.206	1.983	0.040
Carbohydrate metabolism						
Pyruvic acid	7.47	174	767	1.647	0.085	0.007
Lactic acid	7.62	117	970	2.085	0.611	0.050
Ribose	15.86	103	958	1.539	0.740	0.035
Fructose2,6-biphosphatedegrprod	21.26	211	562	1.237	5.659	0.000
Citric acid	17.43	273	633	1.435	6.798	0.003
Succinic acid	11.33	147	882	1.896	1.873	0.011
Biogenic amines						
Spermidine	21.68	144	574	1.741	0.235	0.003
Putrescine	16.60	174	949	2.020	0.295	0.021
Malonamide	14.41	118	253	2.043	0.394	0.011
Bis(2-hydroxypropyl) amine	11.52	196	206	1.979	0.591	0.008
Urea cycle						
Citrulline	17.51	157	666	2.062	0.516	0.001

All different metabolites listed here are those VIP > 1 and *p* value < 0.05

**Table 2:** Different metabolites content in the rumen of dairy cows fed SARA induced diet (SAID) versus cows fed SARA induced diet with thiamine supplementation (SAID + T)

Metabolites	RT	Mass	Similarity	VIP	Fold change	*p* value
Amino acids						
Glycine	11.22	174	944	1.602	2.080	0.021
Valine	9.92	144	958	1.441	2.355	0.040
Organic acids						
Pelargonic acid	11.99	215	933	1.496	1.294	0.039
Oxamic acid	10.57	147	572	1.523	1.405	0.048
Caprylic acid	10.66	143	700	1.128	2.166	0.035
Carbohydrate metabolism						
Pyruvic acid	7.47	174	767	1.066	4.917	0.013
Lactic acid	7.62	117	970	2.454	2.069	0.031
Tagatose	17.72	103	633	1.555	5.517	0.003
Lactose	25.33	204	848	1.008	0.351	0.029
Succinate semialdehyde	9.54	132	267	2.142	0.442	0.005
Biogenic amines						
Indole-3-acetamide	19.05	318	233	1.425	2.283	0.050
Bis(2-hydroxypropyl)amine	11.52	196	206	1.192	2.408	0.008
Spermidine	21.68	144	574	1.492	2.501	0.023

Compared with CON treatment, pyruvate, lactate and ribose were significantly increased in SAID feeding; fructose2, 6-biphosphatedegrprod, citric acid and succinic acid were significantly decreased in SAID feeding. Remarkable alteration in the content of purine metabolites of SAID feeding compared with CON was observed in this study. Hypoxanthine and xanthine were significantly decreased in SAID treatment. Inversely the content of some biogenic amines such as spermidine, putrescine and malonamide were all significantly increased after feeding SAID diet. Compared with SAID treatment, pyruvate and lactate, spermidine, indole-3-acetamide and bis (2-hydroxypropyl) amine were significantly decreased in SAID + T treatment. Pyruvate, lactate, bis(2-hydroxypropyl) amine and spermidine were the main metabolites that significantly increased in SAID treatment compared to CON treatment and significantly decreased after thiamine supplementation.

**Fig. 3:**
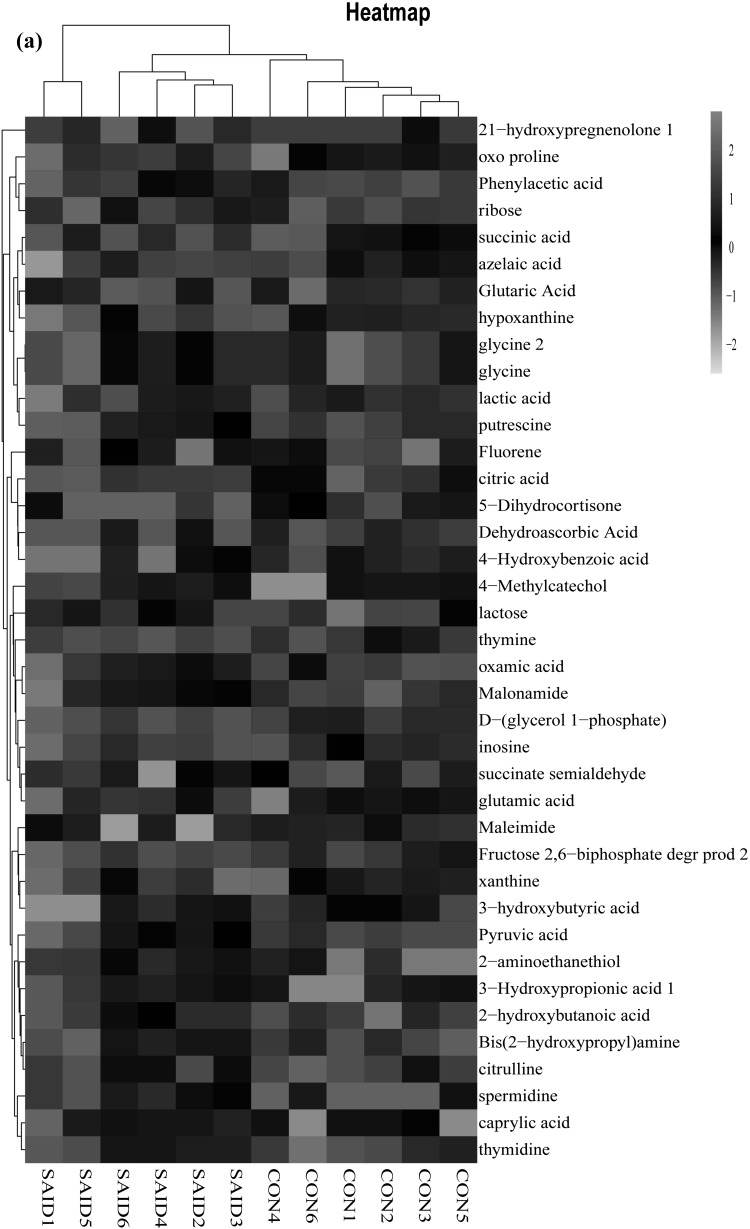

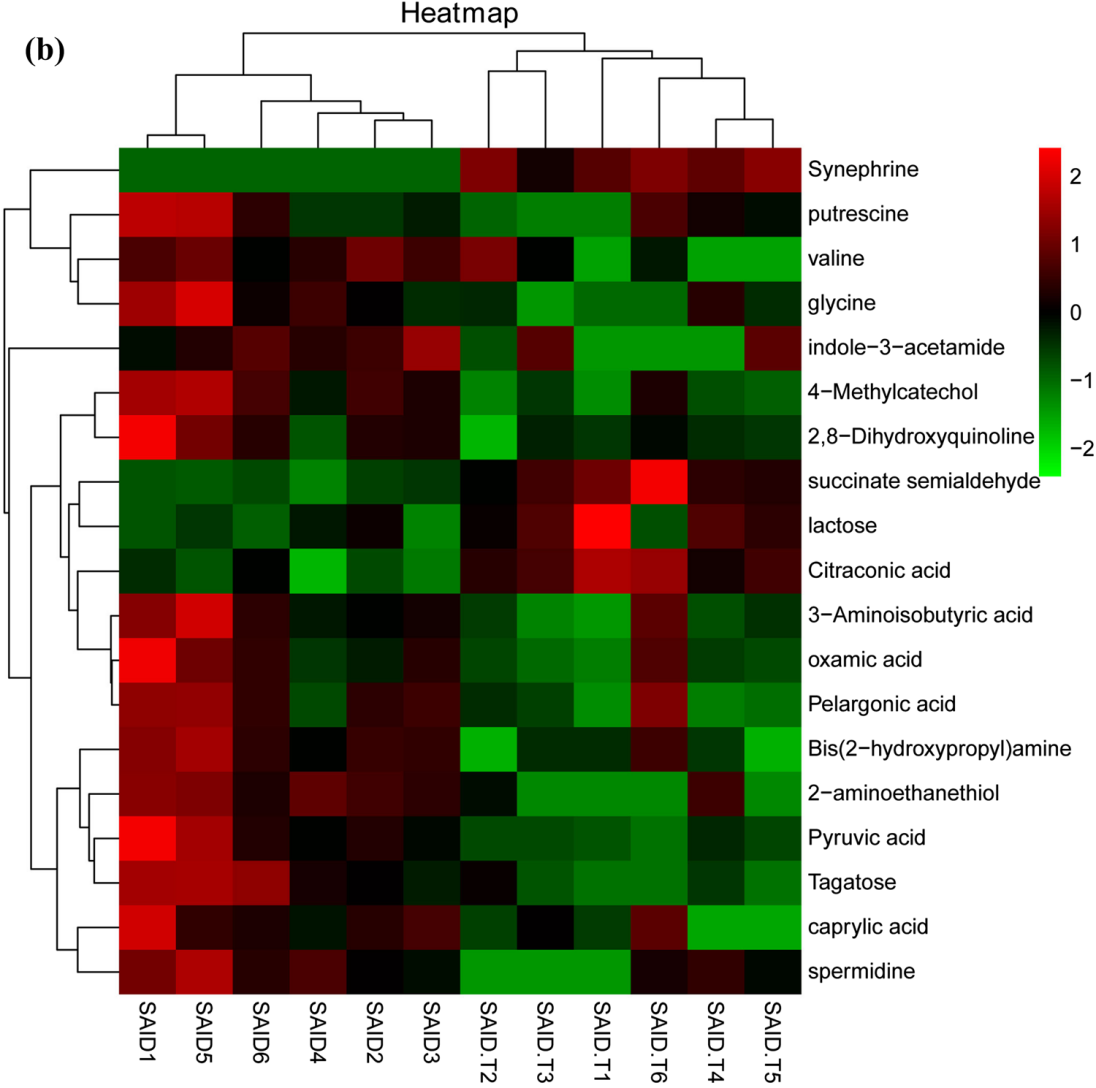
Hierarchical clustering analysis (HCA) and heat maps for different metabolites from the three treatments. **a** HCA of metabolites from cows (n = 6) fed control diet (CON) versus cows fed SARA induced diet (SAID); **b** HCA of metabolites from cows (n = 6) fed SAID versus cows (n = 6) fed SARA induced diet with thiamine supplementation (SAID + T). Rows represent metabolites and columns represent samples. Cells were colored based on the signal intensity measured in rumen, light red represented high rumen levels while green showed low signal intensity and black cells showing the intermediate level

HCA and heat map were used for further understanding of how ruminal metabolites changed in response to the SAID diet and thiamine supplementation. Results were presented in Fig. 3. As shown in Fig. 3a, compared with CON treatment, metabolites that significantly decreased or increased in SAID treatment were separated clearly. At the lower part of the figure, two significant different subclusters were located. One subcluster consists of significantly downregulated metabolites in SAID treatment compared with CON, such as glutamic acid, xanthine and inosine. The other subcluster consisted of nine significantly upregulated metabolites in SAID treatment, such as spermidine, thymidine and pyruvic acid. Figure 3b represented SAID versus SAID + T. Metabolites that significantly decreased in SAID treatment compared with SAID + T treatment or significantly increased in in SAID treatment compared with SAID + T treatment could be separated clearly. HCA revealed metabolites that significantly increased in SAID treatment compared with SAID + T treatment mainly gathered into two subclusters. One consisted of putrescine, valine and glycine; the other consisted of pyruvate, tagatose and spermindine. Metabolites that significantly decreased in SAID treatment compared with SAID + T treatment main gathered into one subcluster, which consisted of lactose, succinate semialdehyde and citraconic acid.

### Pathway analysis

Functional analysis of pathways related to different metabolites was conducted using KEGG. Results of significantly changed pathways are shown in Table 3. Purine metabolism, carbon metabolism, biosynthesis of amino acids, pyruvate metabolism, glutathione metabolism, protein digestion and absorption and thiamine metabolism were detected to be the significantly changed pathways between CON and SAID. Carbon metabolism, biosynthesis of amino acids, protein digestion and absorption, thiamine metabolism and glutathione metabolism were the significantly changed pathways between SAID and SAID + T.

**Table 3:** Metabolic pathways and significant different metabolites that enriched in the pathways of dairy cows fed a control diet (CON) versus cows fed SARA induced diet (SAID)and cows fed SARA induced diet (SAID) versus cows fed SARA induced diet with thiamine supplementation (SAID + T)

Group	Metabolic pathways	Metabolites
CON versus SAID	Purine metabolism—Bos taurus (cow) (6)	Glycine, sulfate, hypoxanthine, inosine, xanthine, guanosine
	Carbon metabolism—Bos taurus (cow) (6)	Pyruvate, l-glutamate, glycine, succinate, citrate, 3-hydroxypropanoate
	Glutathione metabolism—Bos taurus (cow) (6)	l-Glutamate, glycine, putrescine, spermidine, pidolic acid, dehydroascorbate
	Biosynthesis of amino acids—Bos taurus (cow) (5)	Pyruvate, l-glutamate, glycine, citrate, l-citrulline
	Protein digestion and absorption—Bos taurus (cow) (4)	l-Glutamate, glycine, putrescine, 4-cresol
	(TCA cycle)—Bos taurus (cow) (3)	Pyruvate, succinate, citrate
	Fatty acid biosynthesis—Bos taurus (cow) (2)	Decanoic acid, octanoic acid
	Thiamine metabolism—Bos taurus (cow) (2)	Pyruvate, glycine
SAID versus SAID + T	Carbon metabolism—Bos taurus (cow) (3)	Pyruvate, glycine, succinate semialdehyde
	Valine, leucine and isoleucine biosynthesis—Bos taurus (cow) (3)	Pyruvate, l-valine, 2-methylmaleate
	Biosynthesis of amino acids—Bos taurus (cow) (3)	Pyruvate, glycine, l-valine
	Protein digestion and absorption—Bos taurus (cow) (2)	Glycine, l-valine
	Thiamine metabolism—Bos taurus (cow) (2)	Pyruvate, glycine
	Glutathione metabolism—Bos taurus (cow) (2)	Glycine, spermidine

Numbers in “()”represents metabolites enriched in the corresponding pathways

**Fig. 4:**
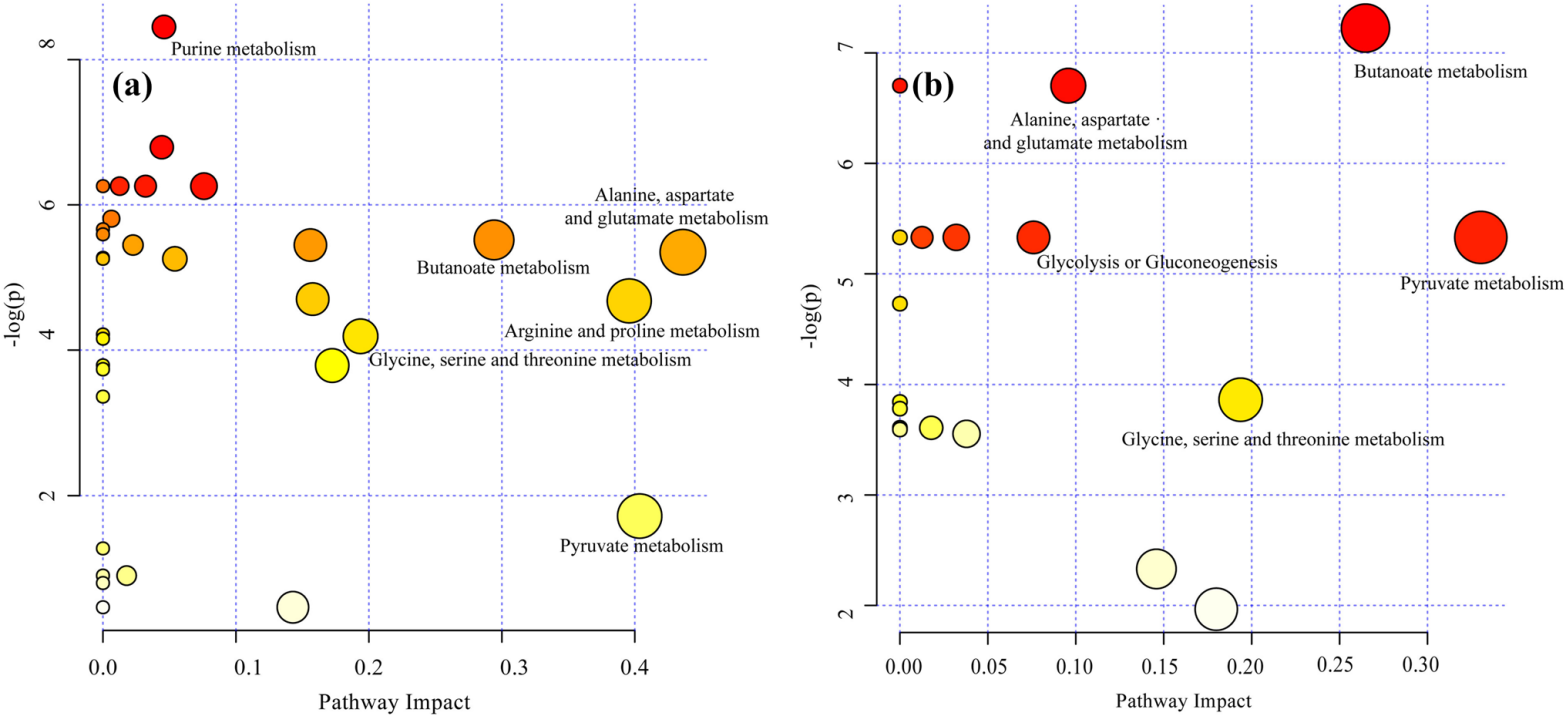
Metabolome view map of the differentially expressed metabolites from the three treatments. **a** Metabolome view map of metabolites from cows (n = 6) fed control diet (CON) versus cows fed SARA induced diet (SAID); **b** metabolome view map of metabolites from cows (n = 6) fed SAID versus cows (n = 6) fed SARA induced diet with thiamine supplementation (SAID + T). X-axis represents pathway impact and Y-axis represents *p* value. The larger size of circle indicates more metabolites enriched in that pathway and the larger abscissa indicates higher pathway impact values. The darker color indicates the smaller *p* values

Based on the different pathways between treatments, pathway topology analysis was conducted. Results are shown in Fig. 4. Based on the pathway impact values, Fig. 4a showed that 6 main metabolic pathways of CON versus SAID were enriched which included arginine and proline metabolism; alanine, aspartate and glutamate metabolism; purine metabolism; glycine, serine and threonine metabolism; butanoate metabolism and pyruvate metabolism. Figure 4b showed that five main metabolic pathways of SAID versus SAID + T were showed such as glycolysis or gluconecogenesis; glycine, serine and threonine metabolism; alanine, aspartate and glutamate metabolism; butanoate metabolism and pyruvate metabolism. Among these metabolic pathways, pyruvate metabolism has the largest impact following thiamine supplementation.

## Discussion

### Effects of thiamine supplementation on carbohydrate metabolism

Based on the present study, we found that thiamine supplementation in SAID diet significantly increased the content of lactose, acetate and succinates; decreased pyruvate, lactate and propionate in ruminal fluid compared with SAID diet. These metabolites are involved in carbohydrate metabolism. In the rumen, dietary carbohydrates are usually converted to pyruvate and acetyl-CoA by microorganisms through the glycolytic pathway and pentose phosphate pathway, and finally acetyl-CoA are mainly metabolized to lactate, VFAs, and a small amount of carbon dioxide and methane (Friggens et al. 1998). Since SAID diet contains a high level of starch, many previous studies demonstrated that feeding a high level starch diet led to disorders of carbohydrate metabolism, causingsignificant increases of lactate and propionate and decreases of acetate (Reddy et al. 2008; Ametaj et al. 2010; Pan et al. 2016). However, few studies reported the significant increased level of pyruvate caused by high-concentrate diets.

In the present study, pyruvate was detected to significantly increase in SAID compared with CON and significantly decrease after thiamine supplementation. Pathway impact analysis indicated that pyruvate metabolism hold the largest pathway impact after thiamine supplementation. The possible reason for the pyruvate accumulation was that high-concentrate diets increased rumen fluid concentrations of glucose (Saleem et al. 2012). In ruminal conditions, pyruvate is the degradation product of glucose through the glycolytic pathway. The increasing glucose promotes the production of pyruvate in the rumen. On the other hand, pyruvate can be converted to lactate by lactate dehydrogenase (LDH) (Chen et al. 2016) or converted to acetyl coenzyme-A and formate by pyruvate formate-lyase (PFL) (Asanuma and Hino 2000), then acetyl-CoA is converted either to acetate or ethanol. Under anaerobic condition, PFL is a central enzyme to degrade pyruvate to acetyl-CoA and the enzyme comprises the cofactors S-adenosylmethionine and thiamine diphosphate (TPP) (Knappe et al. 1969). As the cofactor of PFL, thiamine content was detected significantly decreased when feeding SAID diet (Karapinar et al. 2008; Pan et al. 2016). As a result, the conversion of pyruvate to acetyl-CoA was restrained, and the superfluous pyruvate were then converted to lactate by lactate-producing bacteria such as *S. bovis* (Asanuma and Hino 2000) through LDH, which led to accumulation of lactate and SARA aggravation in dairy cows. Thiamine supplementation could increase the ruminal content of thiamine and TPP, and then improve the activity of PFL to catalyze pyruvate to produce more acetyl-CoA and less lactate, therefore leading to a significant decrease in pyruvate and lactate following thiamine supplementation.

In the present study, succinates were decreased in the SAID treatment compared with the other two treatments. This result was in alignment with the results of Saleem et al. (2012), who found that when dairy cows were fed 45% grain diet, the ruminal succinates content were significantly lower than cows fed 30% grain diet. In rumen, succinates can be produced by many microorganisms, such as *Anaerobiospirillum succiniciproducens* and *Mannheimia succiniciproducens* (Beauprez et al. 2010). However, succinate does not accumulate in the rumen because it is rapidly utilized by *Succiniclasticum* or metabolized to propionate through treatment by propionate-producing bacteria (Kennedy et al. 1991). Our previous study found *Succiniclasticum*, as the primary succinate utilizing bacteria, accounted for 12.45% of total bacterial community from high-grain treatment and increased significantly compared with CON treatment (Pan et al. 2017a). The increasing abundance of *Succiniclasticum* may partially explain the decreased succinates under high-grain feeding. However, the proportions of *Succiniclasticum* was reduced with thiamine supplementation (Pan et al. 2017a), as a result, the ruminal succinate content was increased in SAID + T treatment.

### Effects of thiamine supplementation on amino acids metabolism

In the present study, valine and glycine were significantly decreased after thiamine supplementation compared with SAID. In ruminal conditions, pyruvate and isobutyrate are the main substrates for the biogenesis of valine (Allison and Peel 1971). Thiamine supplementation in SAID decreased rumen isobutyrate (Pan et al. 2016) and pyruvate content which may result in the decreased valine content. Glycine was positive correlated with thiamine metabolism. Previous studies indicated that in bacteria, the thiazole moiety of thiamine was synthesized from glycine, cysteine and deoxy-d-xylulose 5-phosphate (Linnett and Walker 1968; Begley et al. 1999; Settembre et al. 2003). The reason for the accumulation of glycine in SAID might be that, glycine was the substrate of thiamine synthesis and thiamine could be synthesized by *Bacteroidetes, Fibrobacter*, and *Pyramidobacter* in rumen (Pan et al. 2017a). Feeding SAID caused the decrease of *Bacteroidetes, Fibrobacter*, and *Pyramidobacter* (Pan et al. 2017a), thiamine biosynthesis was hindered which led to the accumulation of glycine. Thiamine supplementation could increase rumen *Bacteroidetes, Fibrobacter*, and *Pyramidobacter* content, improved the biosynthesis of thiamine, therefore, ruminal glycine content decreased.

### Effects of thiamine Supplementation on biogenic amines

Biogenic amines are found at low levels in rumen fluid of healthy animals, but will considerably increase when sheep or cattle are fed excessive rapidly fermentable carbohydrates (Zhang et al. 2014). Similarly, we found that biogenic amines including putrescine and spermidine increased in SAID compared with CON. These results were in line with Wang et al. (2013), who found that the concentration of putrescine in ruminal fluid of SARA cows were increased (*p* < 0.001). The increasing biogenic amines could be explained by the lower pH under SAID feeding. Since biogenic amines are produced from decarboxylation of amino acids by decarboxylase in bacteria (Hill and Mangan 1964), and the decarboxylase activity would be enhanced when pH decreased below 5.5 (Gale 1940; Hill and Mangan 1964). The higher amino acid decarboxylase activity would catalyze amino acids to form more corresponding amines. Thiamine supplementation increased the rumen pH in dairy cows (Pan et al. 2016), as a result, the activity of microbial amino acid decarboxylase was reduced and therefore the content of ruminal biogenic amines decreased.

On the other hand, feeding SAID altered rumen microbial population, caused gram-negative bacterium death and increased concentrations of lipopolysaccharide (LPS) in rumen fluid (Khafipour et al. 2009; Pan et al. 2017b). The increased LPS could alter epithelial barrier function, which changes transepithelial electrolyte transport and increases passive permeability of the epithelium to small and large molecules (Khafipour et al. 2009). Destruction of epithelial barrier function during SAID feeding may hinder the absorption of ruminal biogenic amines into blood and result in the accumulation of biogenic amines (Aschenbach and Gäbel 2000). Thiamine could suppress TLR4-mediated NFκB signaling pathways to attenuate the epithelial inflammation during SAID feeding to promote the absorption of ruminal biogenic amines into blood (Pan et al. 2017b). Therefore, ruminal biogenic amines decreased after thiamine supplementation.

## Conclusion

Our data revealed that SAID feeding increased the content of ruminal VFAs, pyruvate, lactic acid and biogenic amines. Accumulation of these metabolites decreased the ruminal pH and led to SARA. Thiamine supplementation could attenuate high-concentrate diet induced SARA by increasing pyruvate formate-lyase activity to promote pyruvate to generate acetyl-CoA and inhibit lactate generation. In addition, the content of biogenic amines was decreased by thiamine supplementation, which alleviated the ruminal epithelial inflammatory response during SARA challenge to some extent. Overall, our findings update understanding of thiamine’s function on ruminal metabolism regulation and provide new strategies to improve dairy cows’ health under high concentrate feeding pattern.

## Electronic supplementary material

Below is the link to the electronic supplementary material.

Supplementary material 1 (ZIP 5089 KB)

## References

[CR1] Allison MJ, Peel JL (1971). The biosynthesis of valine from isobutyrate by *Peptostreptococcus elsdenii* and *Bacteroides ruminicola*. The Biochemical Journal.

[CR2] Ametaj BN, Zebeli Q, Saleem F, Psychogios N, Lewis MJ, Dunn SM, Xia J, Wishart DS (2010). Metabolomics reveals unhealthy alterations in rumen metabolism with increased proportion of cereal grain in the diet of dairy cows. Metabolomics.

[CR3] Asanuma N, Hino T (2000). Effects of pH and energy supply on activity and amount of pyruvate formate-lyase in *Streptococcus bovis*. Applied and Environmental Microbiology.

[CR4] Aschenbach JR, Gäbel G (2000). Effect and absorption of histamine in sheep rumen: Significance of acidotic epithelial damage. Journal of Animal Science.

[CR5] Beauprez JJ, De Mey M, Soetaert WK (2010). Microbial succinic acid production: Natural versus metabolic engineered producers. Process Biochemistry.

[CR6] Begley TP, Downs DM, Ealick SE, McLafferty FW, Van Loon APGM, Taylor S, Campobasso N, Chiu H-J, Kinsland C, Reddick JJ (1999). Thiamin biosynthesis in prokaryotes. Archives of Microbiology.

[CR7] Chen L, Luo Y, Wang H, Liu S, Shen Y, Wang M, Müller V (2016). Effects of glucose and starch on lactate production by newly isolated *Streptococcus bovis* S1 from Saanen Goats. Applied and Environmental Microbiology.

[CR8] Enemark JM (2008). The monitoring, prevention and treatment of sub-acute ruminal acidosis (SARA): A review. Veterinary Journal.

[CR9] Friggens NC, Emmans GC, Kyriazakis I, Oldham JD, Lewis M (1998). Feed intake relative to stage of lactation for dairy cows consuming total mixed diets with a high or low ratio of concentrate to forage. Journal of Dairy Science.

[CR10] Gale EF (1940). The production of amines by bacteria: The decarboxylation of amino-acids by strains of *Bacterium coli*. The Biochemical Journal.

[CR11] Hill KJ, Mangan JL (1964). The formation and distribution of methylamine in the ruminant digestive tract. The Biochemical Journal.

[CR12] Hua C, Tian J, Tian P, Cong R, Luo Y, Geng Y, Tao S, Ni Y, Zhao R (2017). Feeding a high concentration diet induces unhealthy alterations in the composition and metabolism of ruminal microbiota and host response in a goat model. Frontiers in Microbiology.

[CR13] Karapinar T, Dabak M, Kizil O, Balikci E (2008). Severe thiamine deficiency in sheep with acute ruminal lactic acidosis. Journal of Veterinary Internal Medicine.

[CR14] Kennedy DG, Young PB, McCaughey WJ, Kennedy S, Blanchflower WJ (1991). Rumen succinate production may ameliorate the effects of cobalt-vitamin B-12 deficiency on methylmalonyl CoA mutase in sheep. The Journal of Nutrition.

[CR15] Khafipour E, Li S, Plaizier JC, Krause DO (2009). Rumen microbiome composition determined using two nutritional models of subacute ruminal acidosis. Applied and Environmental Microbiology.

[CR16] Knappe J, Schacht J, Mockel W, Hopner T, Vetter H, Edenharder R (1969). Pyruvate formate-lyase reaction in *Escherichia coli*: The enzymatic system converting an inactive form of the lyase into the catalytically active enzyme. European Journal of Biochemistry.

[CR17] Linnett PE, Walker J (1968). Biosynthesis of thiamine. Incorporation experiments with 14C-labelled substrates and with (15N)glycine in *Saccharomyces cerevisiae*. The Biochemical Journal.

[CR18] McCann JC, Luan S, Cardoso FC, Derakhshani H, Khafipour E, Loor JJ (2016). Induction of subacute ruminal acidosis affects the ruminal microbiome and epithelium. Frontiers in Microbiology.

[CR19] Miller BL, Meiske JC, Goodrich RD (1986). Effects of grain source and concentrate level on B-vitamin production and absorption in steers. Journal of Animal Science.

[CR20] Pan X, Xue F, Nan X, Tang Z, Wang K, Beckers Y, Jiang L, Xiong B (2017). Illumina sequencing approach to characterize thiamine metabolism related bacteria and the impacts of thiamine supplementation on ruminal microbiota in dairy cows fed high-grain diets. Frontiers in Microbiology.

[CR21] Pan XH, Yang L, Beckers Y, Xue FG, Tang ZW, Jiang LS, Xiong BH (2017). Thiamine supplementation facilitates thiamine transporter expression in the rumen epithelium and attenuates high-grain-induced inflammation in low-yielding dairy cows. Journal of Dairy Science.

[CR22] Pan XH, Yang L, Xue FG, Xin HR, Jiang LS, Xiong BH, Beckers Y (2016). Relationship between thiamine and subacute ruminal acidosis induced by a high-grain diet in dairy cows. Journal of Dairy Science.

[CR23] Plaizier JC, Krause DO, Gozho GN, McBride BW (2008). Subacute ruminal acidosis in dairy cows: The physiological causes, incidence and consequences. Veterinary Journal.

[CR24] Reddy G, Altaf M, Naveena BJ, Venkateshwar M, Kumar EV (2008). Amylolytic bacterial lactic acid fermentation - a review. Biotechnology Advances.

[CR25] Saleem F, Ametaj BN, Bouatra S, Mandal R, Zebeli Q, Dunn SM, Wishart DS (2012). A metabolomics approach to uncover the effects of grain diets on rumen health in dairy cows. Journal of Dairy Science.

[CR26] Sato S (2015). Subacute ruminal acidosis (sara) challenge, ruminal condition and cellular immunity in cattle. Japanese Journal of Veterinary Research.

[CR27] Settembre EC, Dorrestein PC, Park JH, Augustine AM, Begley TP, Ealick SE (2015). Structural and mechanistic studies on ThiO, a glycine oxidase essential for thiamin biosynthesis in *Bacillus subtilis*. Biochemistry.

[CR28] Valente TNP, Sampaio CB, Lima EDS, Deminicis BB, Cezário AS, Santos WBRD (2017). Aspects of acidosis in ruminants with a focus on nutrition: A Review. Journal of Agricultural Science.

[CR29] Wang DS, Zhang RY, Zhu WY, Mao SY (2013). Effects of subacute ruminal acidosis challenges on fermentation and biogenic amines in the rumen of dairy cows. Livestock Science.

[CR30] Zhang R, Zhu W, Jiang L, Mao S (2017). Comparative metabolome analysis of ruminal changes in Holstein dairy cows fed low- or high-concentrate diets. Metabolomics.

[CR31] Zhang R, Zhu W, Zhu W, Liu J, Mao S (2014). Effect of dietary forage sources on rumen microbiota, rumen fermentation and biogenic amines in dairy cows. Journal of the Science of Food and Agriculture.

