# Supplementary figures and images for: GC–MS analysis of the ruminal metabolome response to thiamine supplementation during high grain feeding in dairy cows
Source: Metabolomics. 2018 May 8;14(5):67. doi: 10.1007/s11306-018-1362-8 (PMC5940720; doi:10.1007/s11306-018-1362-8)

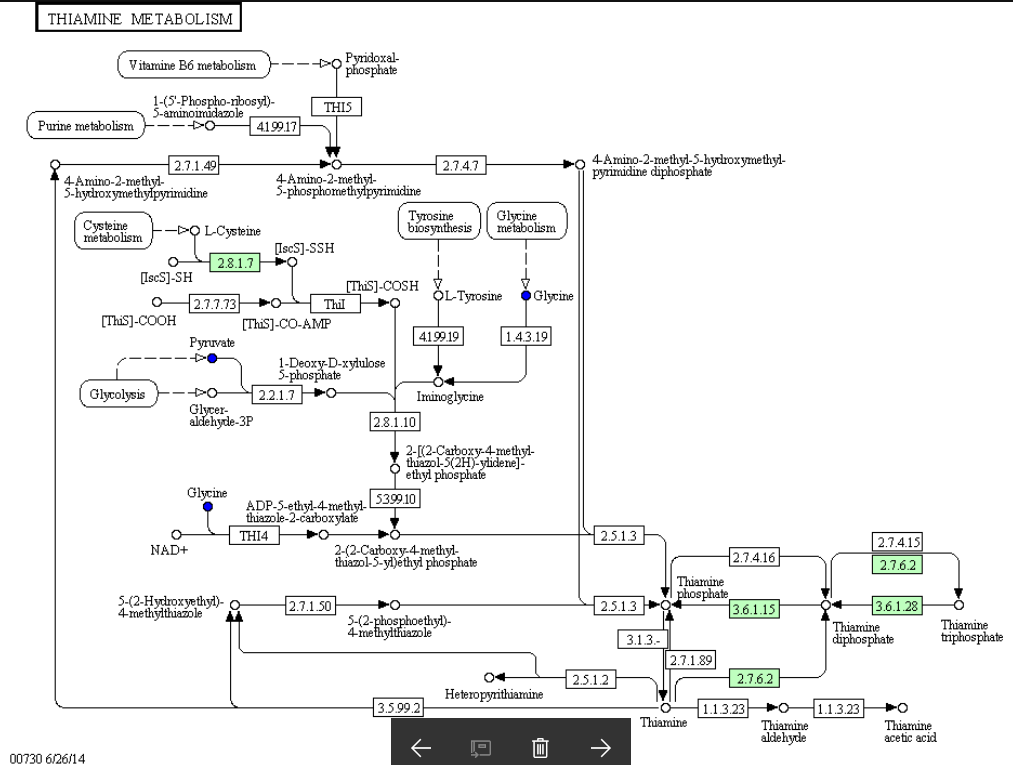

Supplement: Supplementary file 1 — Supplementary material 1 (ZIP 5089 KB) [file 11306_2018_1362_MOESM1_ESM.zip › supplementary informations/supplementary pathways/Thiamine metabolism of SAID+T vs SAID.tiff]

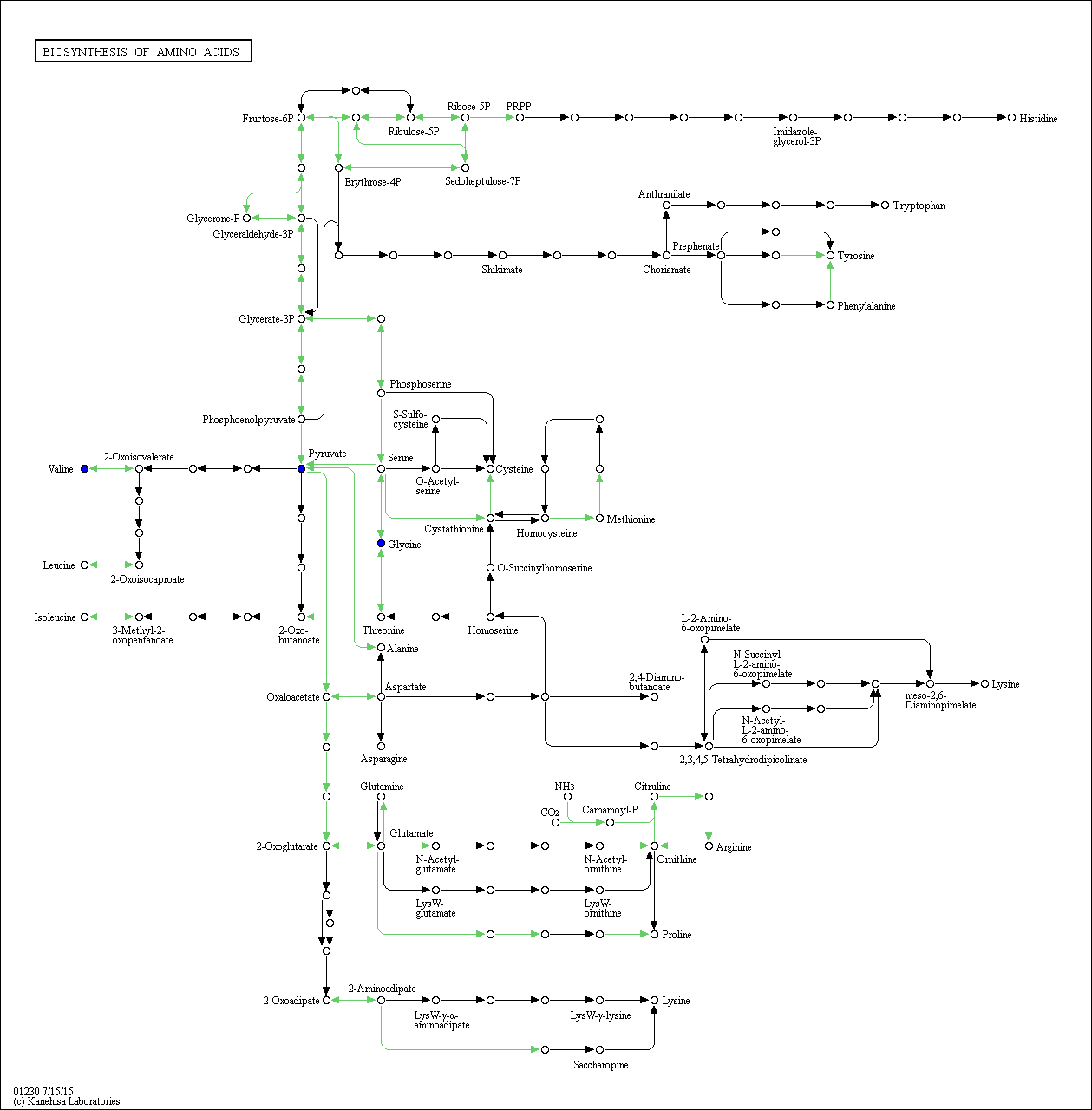

Supplement: Supplementary file 1 — Supplementary material 1 (ZIP 5089 KB) [file 11306_2018_1362_MOESM1_ESM.zip › supplementary informations/supplementary pathways/biogenesis of amino acis of SAID+T vs SAID.TIFF]

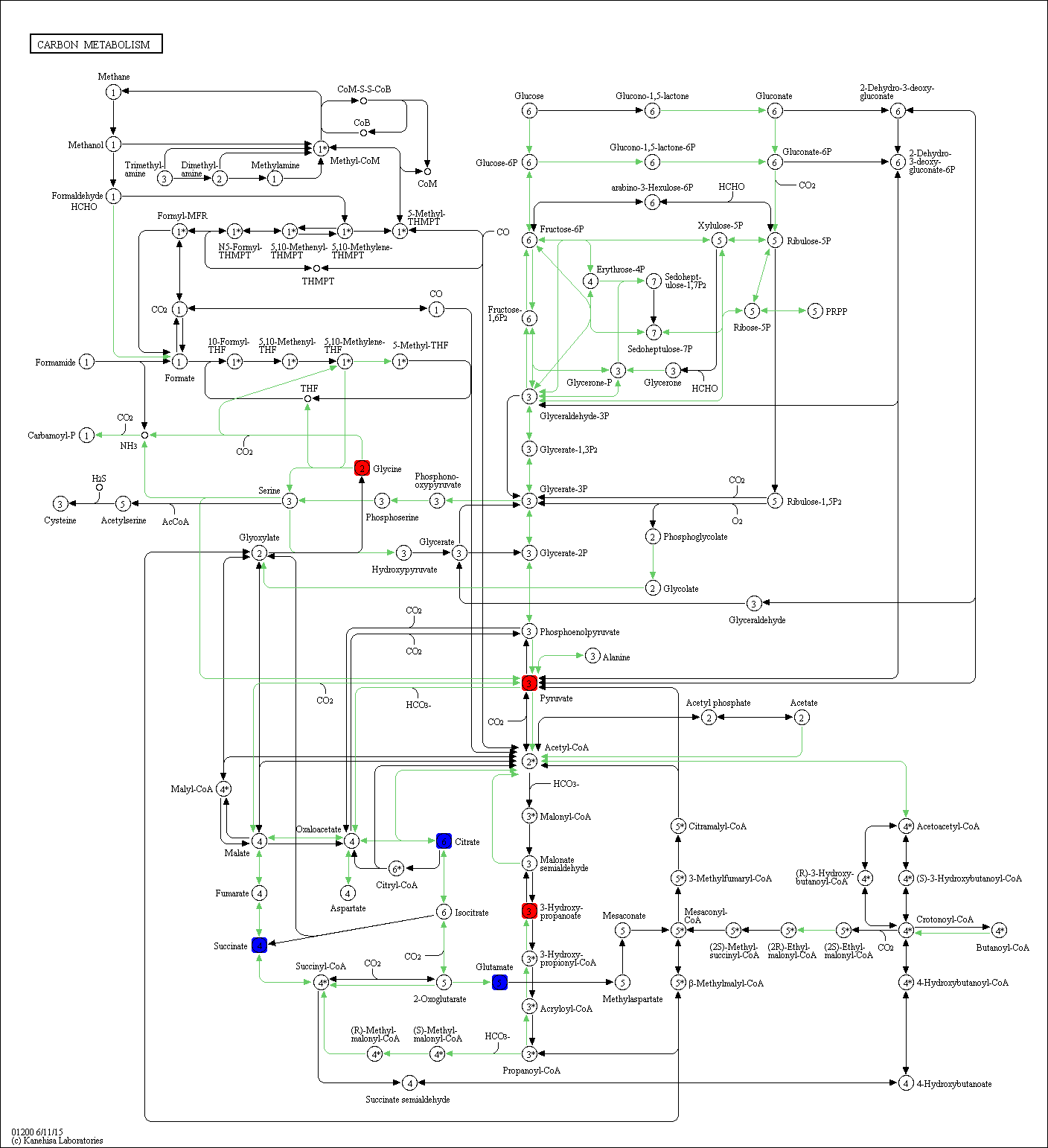

Supplement: Supplementary file 1 — Supplementary material 1 (ZIP 5089 KB) [file 11306_2018_1362_MOESM1_ESM.zip › supplementary informations/supplementary pathways/carbon metabolism of SAID vs CON.TIFF]

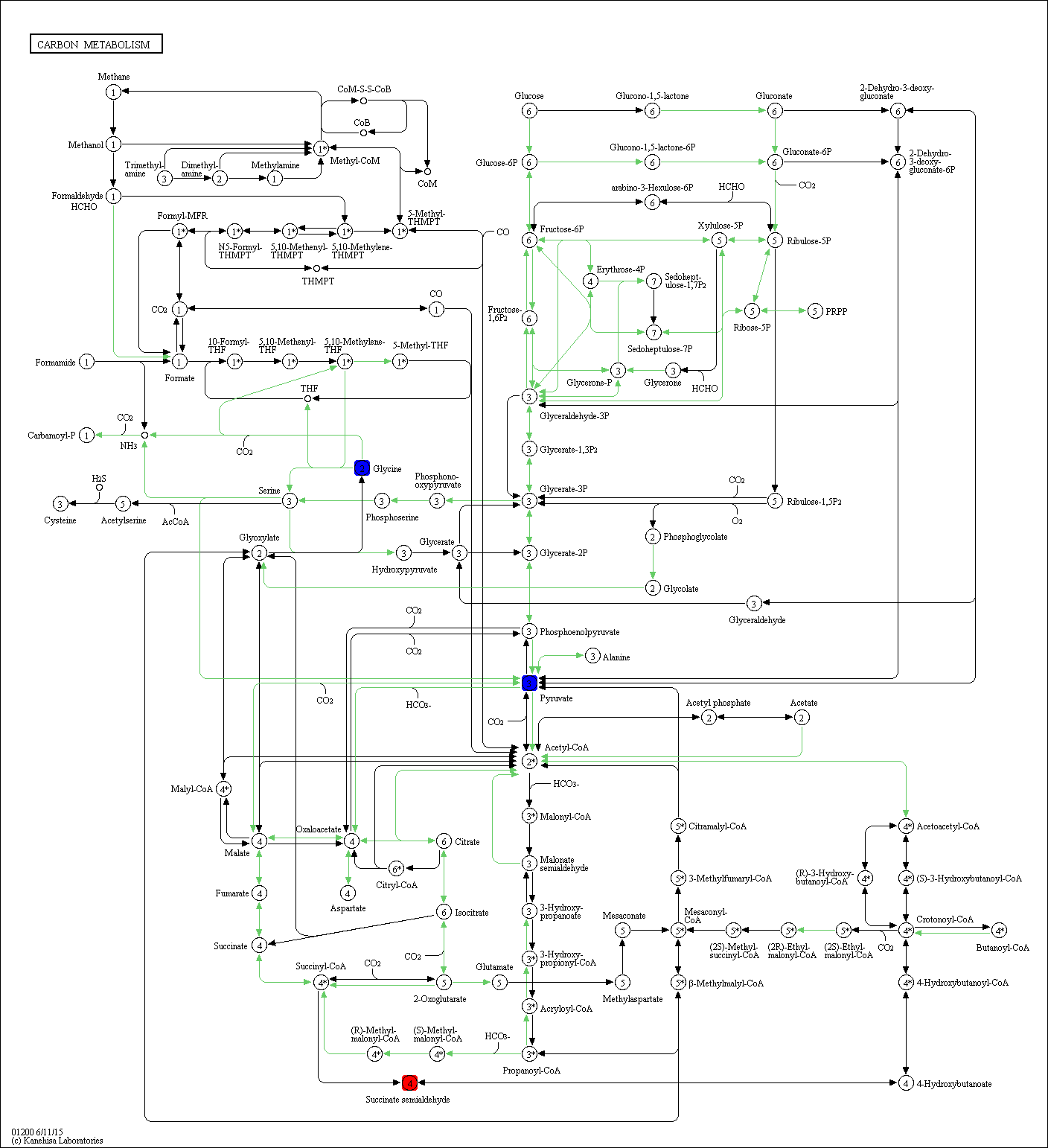

Supplement: Supplementary file 1 — Supplementary material 1 (ZIP 5089 KB) [file 11306_2018_1362_MOESM1_ESM.zip › supplementary informations/supplementary pathways/carbon metabolism of SAID+T vs SAID.TIFF]

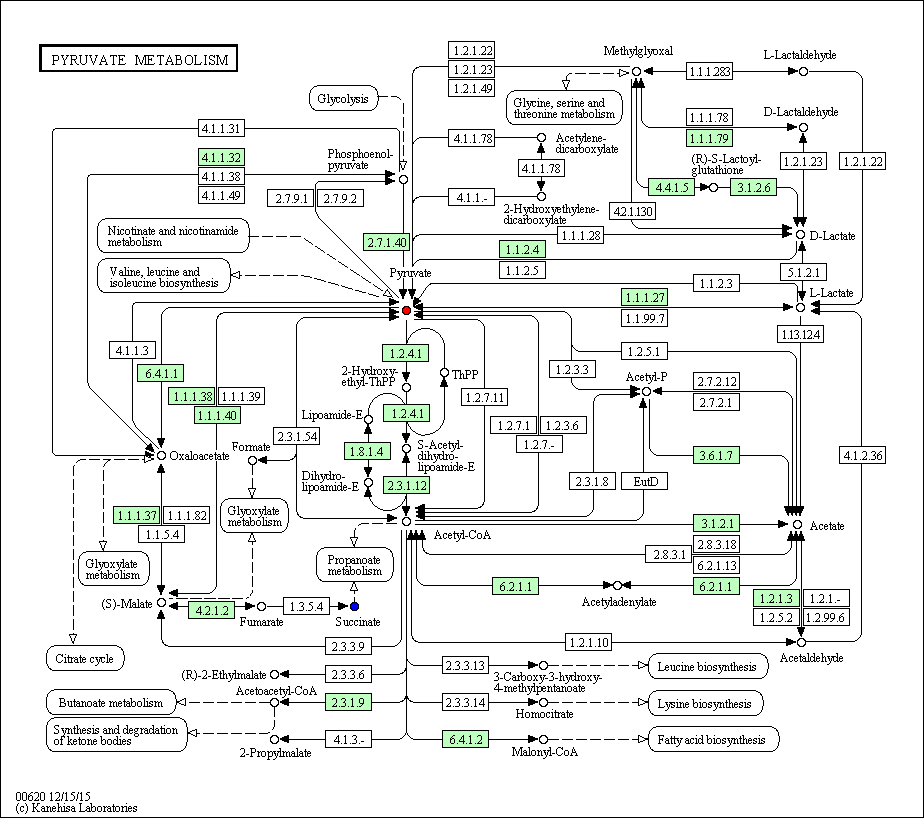

Supplement: Supplementary file 1 — Supplementary material 1 (ZIP 5089 KB) [file 11306_2018_1362_MOESM1_ESM.zip › supplementary informations/supplementary pathways/pyruvate metabolism of SAID vs CON.TIFF]

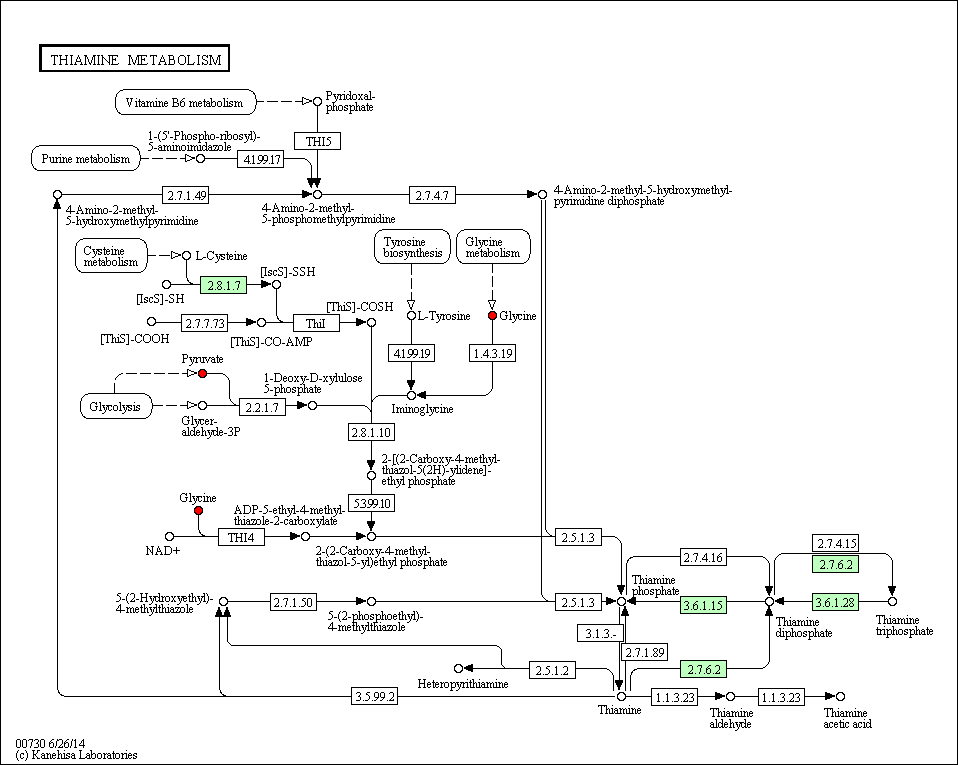

Supplement: Supplementary file 1 — Supplementary material 1 (ZIP 5089 KB) [file 11306_2018_1362_MOESM1_ESM.zip › supplementary informations/supplementary pathways/thiamine metabolism of SAID vs CON.tiff]
